# Positive Effect of Teriparatide on Areal Bone Mineral Density in Young Women with Anorexia Nervosa: A Pilot Study

**DOI:** 10.1007/s00223-020-00791-3

**Published:** 2021-01-08

**Authors:** Gabriella Milos, Hanspeter Moergeli, Cynthia Sob, Doris Wisler, Mariusz Wasila, Daniel Uebelhart, Diana Frey

**Affiliations:** 1 Department of Consultation-Liaison Psychiatry and Psychosomatic Medicine, University Hospital of Zurich, Culmannstr. 8, 8091 Zurich, Switzerland; 2 Clinic for Rheumatology, University Hospital, Zurich, Switzerland; 3 Division of Musculoskeletal, Internal Medicine and Oncological Rehabilitation, Department of Orthopaedics and Traumatology, Centre Hospitalier du Valais Romand (CHVR), Centre Valaisan de Pneumologie (CVP), Hôpital du Valais (HVS), Crans-Montana, Switzerland

**Keywords:** Teriparatide, Osteoporosis, Anorexia nervosa, Cortical and trabecular bone, HRpqCT

## Abstract

The present pilot study investigated the effect of Teriparatide 1–34 rh-PTH (TPT) in young women diagnosed with anorexia nervosa (AN), and markedly compromised Bone Mineral Density (BMD). Patients were included who had (i) very low BMD (defined as *Z*-Score <  − 2.5 or *T*-Score <  − 2.5 if available) in at least one of the assessed localizations (lumbar spine L1–L4, total hip, femoral neck) without any previous fragility fracture; or (ii) low bone mineral density (defined as *Z*-Score <  − 1.5 or *T*-Score <  − 1.5 if available) in at least one of the assessed localizations (lumbar spine L1–L4, total hip, femoral neck) and at least one previous fragility fracture. Ten patients with an age range of 21–33 were recruited and their bone outcome was assessed after 12, 18, and 24 months. After 24 months of TPT treatment, BMD improved by 13.5% in the spine, 5.0% in the femoral neck, and 4.0% in the hip. Radius cortical bone density (− 2.6%) and radius cortical thickness (− 6.4%) decreased significantly, while in tibia there was no significant decrease. Neither in radius nor in tibia a significant change in trabecular bone parameters occurred. During the treatment, the patients’ body weight did not increase significantly. Patients did not experience severe adverse events; only mild side effects were observed. Although these results emerged from a single-arm prospective study, it seems that AN patients with a severely compromised bone situation can benefit from TPT. Larger studies are needed to ascertain the effect of this promising substance.

## Introduction

Anorexia Nervosa (AN) is a psychiatric illness that affects about one percent of women, mostly at a young age [1]. The illness is difficult to treat and has an important tendency to become chronic [2]. Characteristic features of AN are self-induced starvation, weight loss and secondary problems associated with malnutrition; both physical and mental co-morbid conditions are common [3]. Since four decades it is known that low bone mineral density (BMD) is a frequent complication in AN; young patients can indeed present a severe deterioration of BMD, and bone fracture is not unusual [4].

The cause of the loss of bone mass and bone structure in AN is multifactorial; it is the result of hormonal adaptations to the state of starvation. AN—as a form of chronic undernutrition—is characterized by hypogonadotropic hypogonadism, growth hormone resistance, and hypercortisolemia [5].

Weight recovery and long term weight stabilization is the best treatment for low BMD in AN, coupled with a return of normal menses [6]; however, since only 50–60% of women with AN recover even two decades after their diagnosis [7, 8], pharmacological treatment options are needed to reduce the chronic bone loss and the consequent increase in fracture risk.

So far, there is no approved pharmacological treatment to target bone loss in AN [5]. Some pharmacological therapies have been investigated in short term studies in adults with AN, and have demonstrated some efficacy, e.g., bisphosphonates during 12 months [9], insulin-like growth factors (IGF-1) together with oral contraceptives during 9 months [10], and transdermal estradiol replacement during 18 months [11], oral contraceptives, and dehydroepiandrosterone [12], or teriparatide (human parathyroid hormone) during 6 months [13]. Teriparatide (TPT), the recombinant human 1–34 parathyroid hormone, is a bone anabolic agent that is approved for the treatment of postmenopausal osteoporosis in women, hypogonadal osteoporosis in men, and glucocorticoid induced osteoporosis. Since TPTH has a potent anabolic effect on bone, this substance is of high interest in the treatment of low bone mass in AN. A randomized controlled trial reported an elevate increase in spine BMD (6–10%) after 6 months of treatment, compared to placebo in 21 women (mean age 47 ± 1.7 years) with AN [13]. Unlike to this study, we decided to test this substance during a longer time period (24 months) in young women diagnosed with AN and bone deterioration. All patients had very low BMD in at least one of the assessed localizations (lumbar spine L1–L4, total hip, femoral neck) without any previous fragility fracture, or low BMD in at least one of the assessed localizations (lumbar spine L1–L4, total hip, femoral neck) and at least one previous fragility fracture.

BMD was measured with dual energy X-ray absorptiometry (DXA). To capture also changes in the bone microarchitecture due to TPT, High-Resolution peripheral quantitative Computed Tomography (HRpqCT) was also assessed. With the TPT treatment, we expected an increase in the BMD, and positive changes also in the HRpqCT parameters.

## Methods

### Inclusion Criteria

All patients were recruited in the in- and out-patients of the Eating Disorders Centre of the Department of Consultation-Liaison Psychiatry and Psychosomatic Medicine of the University Hospital of Zurich. Female patients aged 18–35 years (closed epiphyses; patients younger than 22 years with closed epiphyses have to be confirmed with forearm X-ray) with anorexia nervosa since at least 12 months before inclusion.

Two bone conditions were analyzed to select the patients to be included in the study: (i) *very low bone mineral density* with or without previous fragility fracture. Very low bone mineral density is defined as a *Z*-Score at screening of lower than − 2.5 (or *T*-Score lower than 2.5 if available) in one or more of the assessed areas (L1–L4, total hip or femoral neck); (ii) *low bone mineral density* with *at least one previous fragility fracture*. Low bone mineral density is defined as *Z*-Score <  − 1.5 (or *T*-Score <  − 1.5 if available) in one or more of the assessed areas (L1–L4, total hip or femoral neck). All participants did agree to practice safe contraception during the overall period of the study and were capable of fully understanding the nature of the study. They did also sign the Informed Consent after being fully informed. All participants were also able to comply with the study protocol.

### Exclusion Criteria

Exclusion criteria were: Metabolic bone diseases other than primary osteoporosis (including hyperparathyroidism, osteomalacia, Paget’s disease of bone), pre-existing hypercalcemia, severe renal impairment (GFR < 30 ml/min), prior external beam or implant radiation therapy to the skeleton, skeletal malignancies or bone metastases, any unknown elevation of serum alkaline phosphatase, severe psychiatric diseases other than AN, drug addiction, HIV positive patients, open epiphysis. Even contraindications to the class of drugs under study, e.g., known hypersensitivity or allergy to a class of drugs or the investigational product. Contraindications for ethical reasons, as determined by the investigator. Receiving or having received specific treatments against osteoporosis (e.g., bisphosphonates, zoledronate, denosumab) except calcium and vitamin D. Previous therapy with calcium and/or vitamin D was allowed if on stable therapy for more than 1 month. The hormone replacement therapy is allowed if a stable dose is given for more than 1 month before screening and even before the use of TPT.

Other exclusion criteria were pregnancy or a breast feeding state, the intention to become pregnant during the course of the study, the lack of safe contraception (safe contraception was defined as follows: Female subjects of childbearing potential, using a medically reliable method of contraception for the entire study duration, such as oral, injectable, or implantable contraceptives, or intrauterine contraceptive devices, or using any other method considered sufficiently reliable by the investigator in individual cases; female subjects who are surgically sterilized/hysterectomized).

Further exclusion conditions were known or suspected non-compliance, drug or alcohol abuse, the inability to follow the procedures of the study (e.g., due to language problems, psychological disorders, dementia or a confusional state of the subject), participation in another study with any investigational drug taken within the 30 days preceding and during the present study; previous enrollment in the current study; enrollment of the investigator, his/her family members, employees and other dependent people.

The evidence of severe hyper- or hypoparathyroidism (PTH > 800 ng/l or < 10 ng/l), hypocalcemia (total calcium < 1.8 mmol/l) or hypercalcemia (total calcium > 2.7 mmol/l) was also an exclusion criteria, as well as any other severe disease that in the opinion of the investigator could lead to metabolic bone disorder or influence fracture rate (e.g., metastatic cancer, osteogenesis imperfecta, severe untreated hypo-/hyperthyroidism, other medications influencing bone metabolism).

### Procedures and Assessments

The diagnosis of AN was made according to DSM IV-TR [14] at the Center for Eating Disorders, Department of Consultation-Liaison Psychiatry and Psychosomatic Medicine, University Hospital of Zurich. All participants were examined by a rheumatologist (DF). Part of the standard rheumatological assessment was a questionnaire on dietary calcium intake. Furthermore, participants completed a questionnaire including socio-demographic data, the history of eating disorder, (under)weight, menstruation, the use of medication, hormonal substitution or contraceptives, as well as the intake of vitamin, mineral, or calcium products. The questionnaire also included questions on the number of hours spent per week participating in sport. Out of 84 pre-screened patients 71 were not eligible for the study mainly because of no low BMD (46%). Of the remaining 13 patients fulfilling inclusion and exclusion criteria three withdrew consent before the beginning of the study. Two patients were non-compliant in administering TPT during the study after 6 and 18 months, respectively. One patient withdrew consent after 12 months. Seven patients concluded the study regularly.

Study participants self-administered 20 µg of TPT once daily sc during 24 months.

### Anthropometric Measurements

Subjects were weighed on an electronic scale in underwear, and their height was measured with a stadiometer. The body mass index (BMI) was calculated using the formula weight in kilograms divided by height in meters squared.

### Sociodemographic, Baseline Characteristics and Safety Laboratory Parameters

All patients were female and had a mean age of 25.4 years at the beginning of the study (see Table 1). The BMI ranged at the baseline from 13.6 to 18.8 kg/m^2^ (mean ± SD: 15.6 ± 1.6), while the minimum lifetime BMI was 12.9 ± 1.4 kg/m^2^. Eight patients (80%) had AN of the restricting type and two AN of the binge-purge type. The average duration of the disorder was 8.8 years. Two patients (20%) had a history of clinical significant fractures. The average age of menarche was 12.8 years. One patient had primary amenorrhea. 80% used sexual hormones with an average intake duration of 39 months (SD 41). All patients used calcium products and vitamin D, the intake of these supplements was not interrupted during the TPT treatment. No patients had osteoporosis specific treatment in the past like, e.g., bisphosphonates, or denosumab.

**Table 1:** Patient characteristics (n = 10). Values presented as mean ± SD unless otherwise noted

Variable	Value	Range
Age, years	25.4 ± 4.4	21–33
Age at menarche, years	12.8 ± 1.7^a^	10–16
Age at AN onset	16.6 ± 2.7	13–21
Duration of the disorder, years	8.8 ± 4.4	4–17
Minimum lifetime BMI, kg/m^2^	12.9 ± 1.4	10.5–16.0
BMI at baseline, kg/m^2^	15.6 ± 1.6	13.6–18.8
Total physical activity, h/week	5.7 ± 3.1	0–8
History of fracture, %	20	
Use of calciuim products^b^, %	100	
Vitamin D use^b^, %	100	
Sexual hormone use^c^, %	80	
Smoking, %	20	
Alcohol use, %	10	
Hashish use, %	0	

^a^*n* = 9, one patient with primary amenorrhea

^b^Calcium 500 mg and vit D 800 UI

^c^8 of 10 patients were under hormonal treatment at the beginning and during the trial. 4 of these 8 patients were treated with hormonal replacement therapy (3 × oral Estradiolvalerat/Norgestrel, 1 × transdermal Estradiol/oral Dydrogesteron), 4 of the 8 patients took contraceptive drugs (1 × Ethinylestradiol/Gestoden, 2 × Ethinylestradiol/Drospirenon, 1 × Ethinylestradiol/Cyproteronacetat).One patient of the study completers and one patient of the dropouts did not have hormonal treatment

Safety laboratory parameters assessed were: Total calcium, calcium correction for albumin, phosphate, creatinine, eGFR, vitamin D, FSH, estradiol, AP bone specific mass, intact parathormone.

### Measurement of Bone Mineral Density, Body Composition and Bone Micro-Architecture

All patients were examined with the following imaging methods:

*Dual-energy X-ray absorptiometry (DXA)* of the lumbar spine (L2–L4), the total proximal hip area and the femoral neck were performed using a Hologic QDR 4500 device (Hologic Corp., Inc., Waltham USA). DXA was used to measure the areal bone density of the lumbar spine (L2–L4), of the non-dominant femoral neck and of the total proximal hip area and it is expressed in grams per square centimeter. In addition, all values were expressed as *T* Scores, as absolute numbers defined in 1994 by WHO (amount of standard deviations below or above the mean bone density value of young white Caucasian women at peak bone mass). The precision of the DXA measurement and the coefficient of variation (CV) at the level of the lumbar spine was 1%, at the hip 1.5%, respectively. The least significant change (LSC at 95% confidence level) was 0.029 g/cm^2^ (CV% 3.04) at lumbar spine, 0.023 g/cm^2^ (CV% 2.71) at hip total, and 0.032 g/cm^2^ (CV% 4.90) at femoral neck. DXA also allows to measure the body composition assessing percent of fat and lean mass (LM) including bone mineral content. The DXA measurements were conducted in the Department of Rheumatology at University Hospital of Zurich.

*High-resolution peripheral quantitative computed tomography (HRpqCT) of the tibia and* of the *non-dominant forearm* using multislice three-dimensional quantitative computer tomography (Scanco™ Medical, Bassersdorf, Switzerland). This method provides to achieve a number of parameters, which describe the microarchitecture of the bone tissue, and to obtain important information about the biomechanical properties of the bone. The measurement protocol included acquisition of a three-dimensional stack of 60 high-resolution CT slices at the most distal end of the radius using a total observation region of 10 mm. Slice thickness was 0.28 mm, pixel matrix 512 × 512 and pixel size 0.17 mm. To obtain cubic voxels, the consecutive cross-sectional slices were measured in steps of 0.17 mm in the axial direction. Measuring parameters are defined as follows: D100, mean entire bone (cortical and trabecular) density of the ultradistal part of the radius in grams hydroxyapatite equivalence per cm^3^ (grHA/cm^3^); Dcomp: bone density of the cortical part of the bone in grHA/cm^3^; C.Th.: absolute thickness of cortical bone in mm; Dtrab: density of the trabecular area of the bone in grHA/cm^3^; Dmeta: density of the subcortical area of the trabecular bone in grHA/cm^3^; Dinn: Density of the central part of the trabecular bone in grHA/cm^3^; Dinn and Dmeta together correspond to the total trabecular bone; BV/TV: relative bone volume as part of the total volume percent; Tb.N.: absolute number of trabecules per mm area; Tb.Th.: mean thickness of bone trabecules in mm; Tb.Sp.: mean separation distance between trabecules in mm. The precision of HRpqCT was < 1% [12, 13, 15, 16] . The HRpqCT were conducted at the Center for Age and Mobility of the Waid Hospital in Zurich.

The study was approved by the Cantonal Ethics Committee of Zurich (KEK-ZH-No. 2012-0297) and by the regulatory Authority Swissmedic (Reference No. 2013DR3141). The trial was also listed in ClinicalTrials.gov (NCT number: NCT01801397). All participants gave their written informed consent before being enrolled into the study.

### Statistical Analysis

The primarily analyzed outcome of the study was defined as the absolute change of the BMD levels in the spine, the total hip and the femoral neck over the course of the study from baseline upon the end of the treatment period with TPT. To perform the intention-to-treat analyses (ITT), linear mixed model analyses including all participants (*N* = 10) were computed with time as a fixed factor. Model estimated marginal means of the outcome measures allowed to compare baseline (0 months) with later assessments (i.e., 12, 18 and 24 months). Models were optimized by selecting a covariance structure for repeated measures that produced the lowest Akaike’s Information Criterion (AIC). For all calculated models, either a heterogeneous first-order autoregressive (ARH1), a first-order factor analytic (FA1), or a scaled identity (ID) covariance structure was appropriate. The threshold for statistical significance was set at *p* ≤ 0.05. Results are also given in % total change from baseline to the end of treatment. Further linear mixed model analyses were performed with secondary outcome parameters. Since a change in BMI, percent fat, and lean mass respectively are potential confounding variables for changes in the outcome measures, we analyzed these variables in the course of time too. Additionally, we computed linear mixed model analyses including these potential confounders as covariates. Each preceding analysis with significant results was repeated with all three confounders; however, including one confounder at a time. In order to complement the mixed model analyses, paired-samples *t*-tests were calculated with primary and secondary outcome parameters in the per-protocol analysis (PP), completers; *n* = 7 comparing baseline with end of treatment values. BMD *Z*-scores of the completers are presented too. To avoid multiple testing, no further analyses with Z-scores were performed. Finally, using again paired-samples t-tests, changes within safety laboratory parameters were analyzed in per-protocol participants. For all analyses, we used IBM SPSS version 25 (IBM Corporation, Armonk, NY, USA).

## Results

**Fig. 1:**
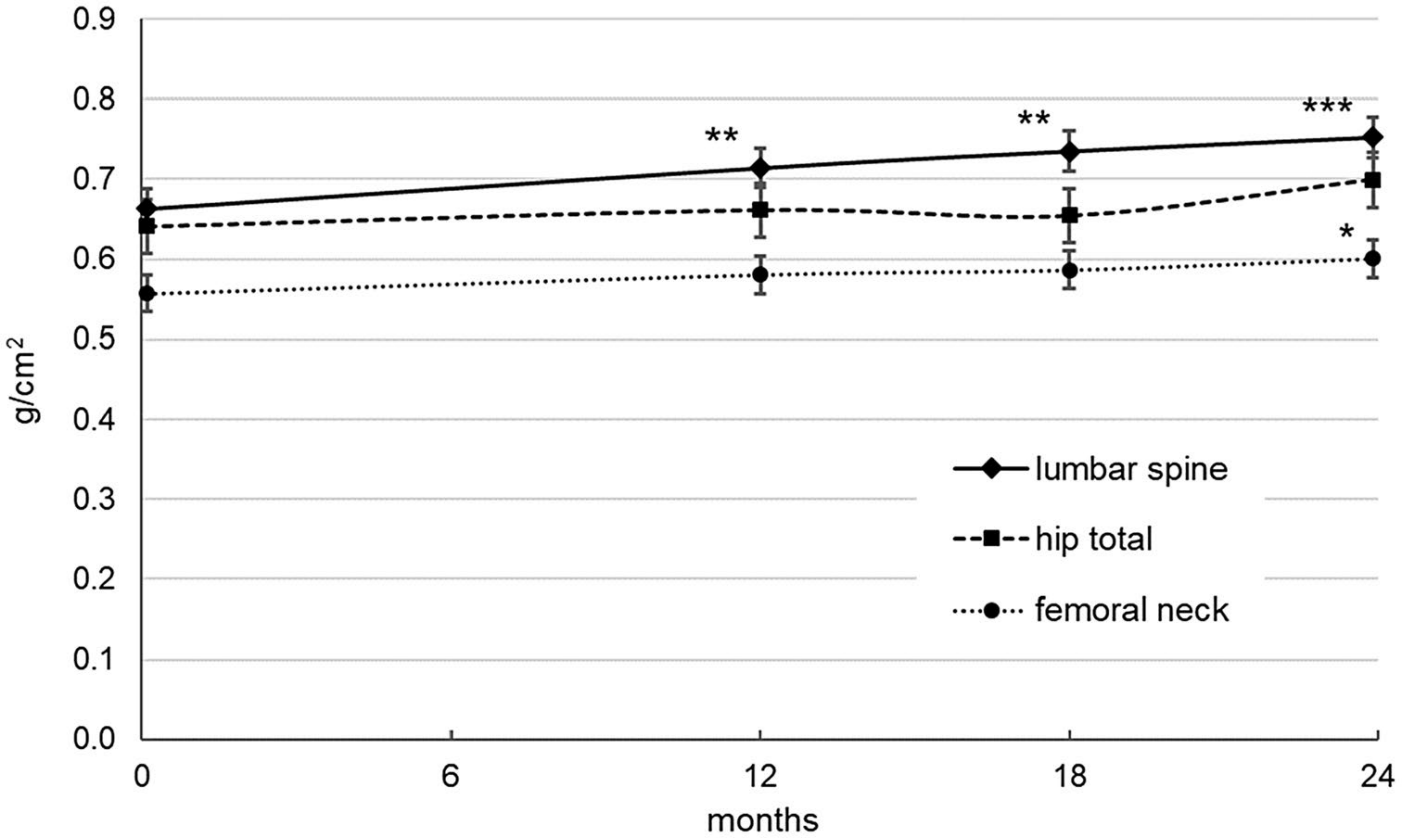
Bone mineral density (g/cm^2^) as measured with Dual-energy X-ray absorptiometry (DXA) of lumbar spine, total hip and femoral neck in the course of time. Model estimated means (± std. error) at baseline, 12, 18 and 24 months. Indicators for significant changes compared to baseline (*p ≤ .05, **p ≤ .01, ***p ≤ .001)

The primary endpoint consisted in testing the effect of TPT treatment during a period of 24 months in the lumbar spine, femoral neck and total hip BMD in patients with severe AN with an age range 21–33, very low bone mineral density and increased bone fragility. The results of the intention-to-treat analysis (ITT), (*n* = 10) are shown in Fig. 1. There was a significant increase in lumbar spine BMD after 12 months (mean difference = 0.051 g/cm^2^, SE = 0.015, *p* < 0.01), 18 months (mean difference = 0.072 g/cm^2^, SE = 0.019, *p* < 0.01) and 24 months (mean difference = 0.090 g/cm^2^, SE = 0.019, *p* < 0.001, total change = 13.5%). For the femoral neck BMD, the treatment showed its effect after 18 (mean difference = 0.026 g/cm^2^, SE = 0.011, *p* < 0.05) and 24 months (mean difference = 0.028 g/cm^2^, SE = 0.009, *p* < 0.05, total change = 5.0%). After 24 months of treatment, the BMD level of the total hip area showed no significant increase (mean difference = 0.026 g/cm^2^, SE = 0.023, *p* = 0.33, total change = 4.0%).

**Fig. 2:**
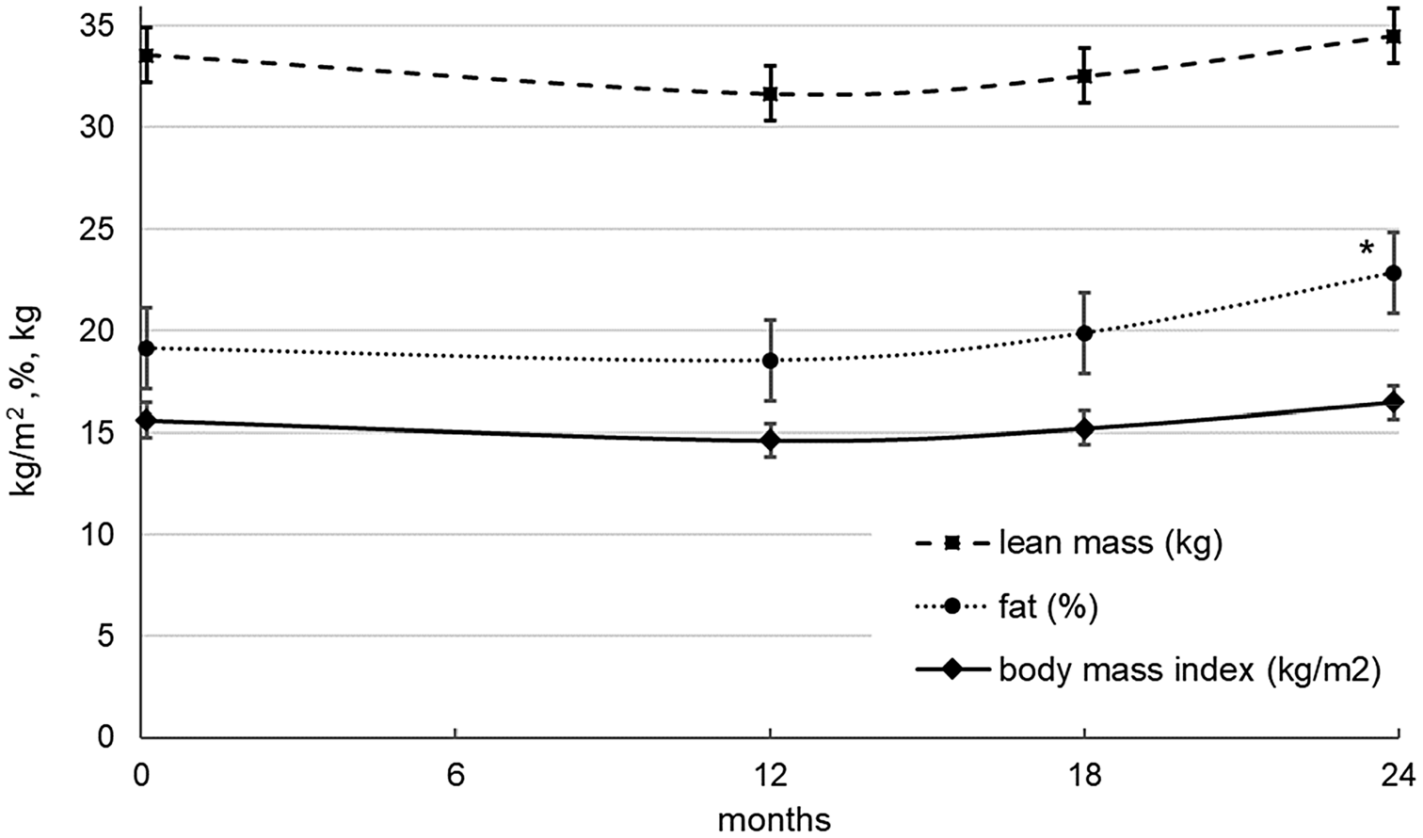
Body mass index (kg/m^2^), percent fat, and lean mass incl. bone mineral content (kg) in the course of time. Model estimated means (± std. error) at baseline, 12, 18 and 24 months. Indicator for significant change compared to baseline (*p ≤ .05, **p ≤ .01, ***p ≤ .001)

Regarding the potential confounders, there was no significant change in BMI from baseline to the end of treatment (mean difference = 0.872 kg/m^2^, SE = 0.466, *p* = 0.11, total change = 5.6%) and in lean mass (mean difference = 0.927 kg, SE = 0.709, *p* = 0.23, total change = 2.8%), while an increase in percent fat occurred (mean difference = 3.74%, SE = 1.60, *p* < 0.05, total change = 19.6%, see Fig. 2). By repeating the mixed model analyses of the primary endpoints under inclusion of the confounders as covariates, the significant results reported above were not essentially altered. The increase in lumbar spine BMD turned out to be still significant at each time point (12 months: *p* < 0.05, 18 months: *p* < 0.01, 24 months: *p* < 0.001). In addition, the model estimated increase in femoral neck BMD was still significant at 18 and 24 months (*p* < 0.05).

**Fig. 3:**
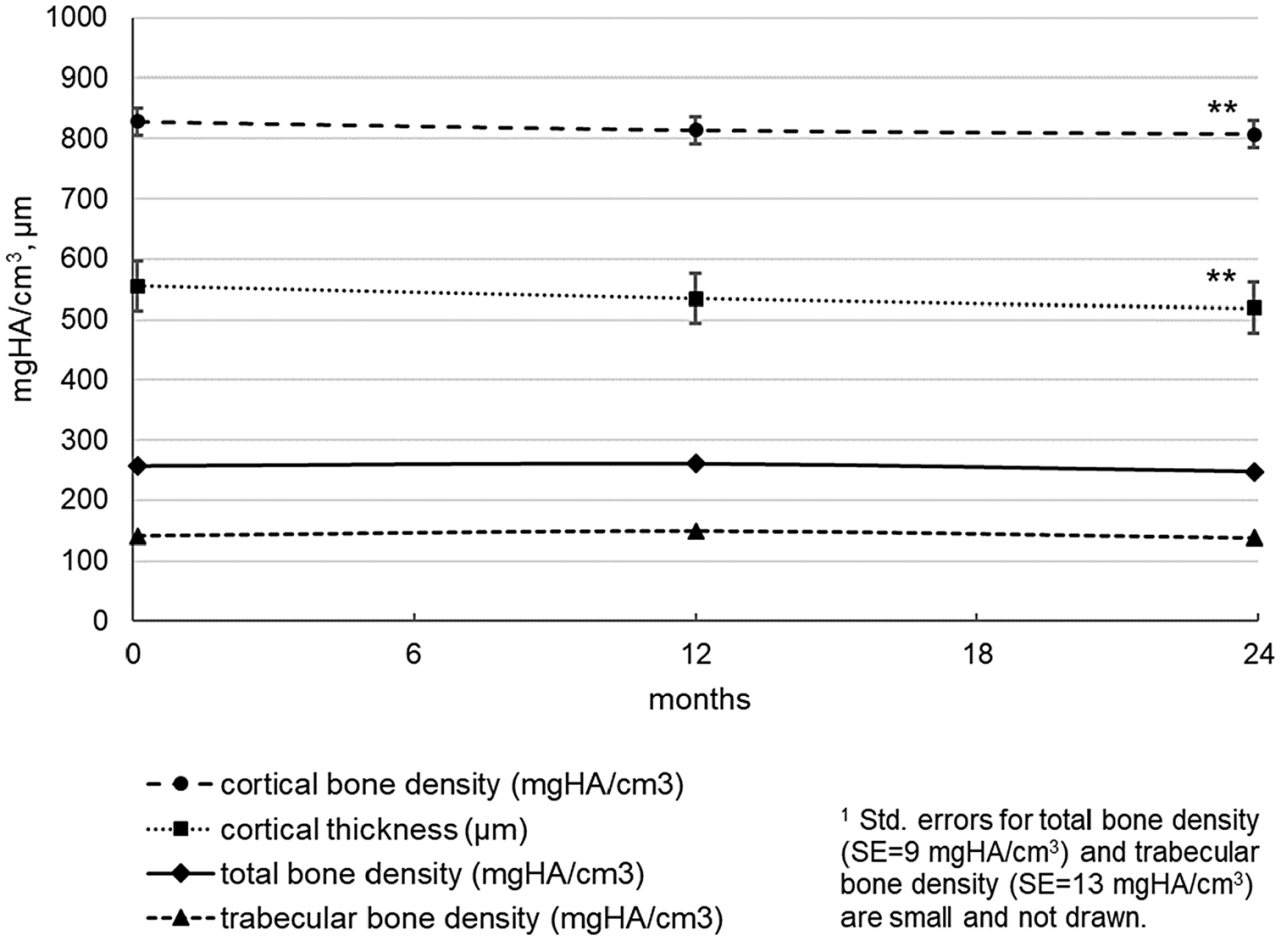
Radius bone parameters as measured with high-resolution peripheral quantitative computed tomography (HRpqCT) in the course of time. Model estimated means (± std. error)^1^ at baseline, 12 and 24 months. Indicators for significant changes compared to baseline (*p ≤ .05, **p ≤ .01, ***p ≤ .001)

The evolution of the HRpqCT parameters on the radius bone is shown in Fig. 3. During the study period of 24 months, radius cortical bone density (mean difference = 21.8 mgHA/cm^3^, SE = 4.77, *p* < 0.01, total change =  − 2.6%) and radius cortical thickness (mean difference = 35.6 μm, SE = 10.8, *p* < 0.01, total change =  − 6.4%) decreased significantly. In radius, no significant change occurred neither in total bone density nor in trabecular bone density. By repeating these analyses under inclusion of the potential confounders (BMI, percent fat, and lean mass), these significant results were not altered, with the exception of the significance level of radius cortical thickness decrease (*p* < 0.05), when controlling for percent fat.

**Fig. 4:**
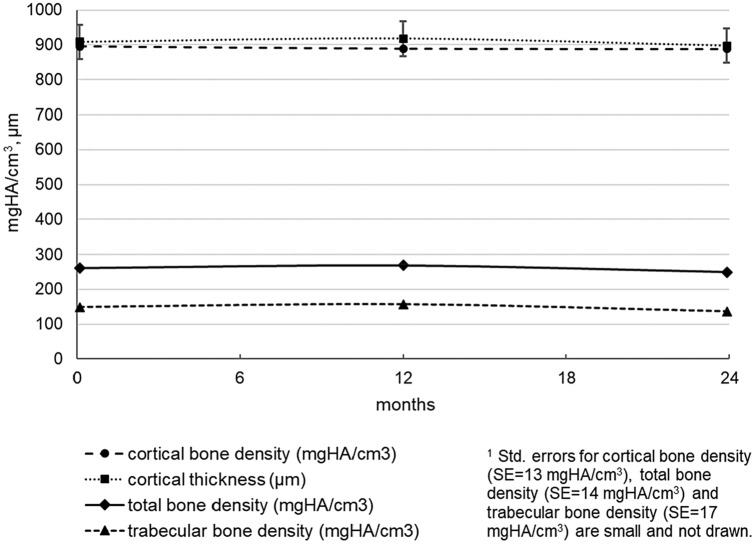
Tibia bone parameters as measured with High-Resolution peripheral quantitative Computed Tomography (HRpqCT) in the course of time. Model estimated means (± std. error)^1^ at baseline, 12 and 24 months. No significant changes compared to baseline (*p* > .05)

With respect to HRpqCT tibia parameters and bone parameters Fig. 4, no significant changes occurred from baseline to the end of the study, e.g., there was a small non-significant change in tibia total bone density at 24 months (mean difference = 12.2 mgHA/cm^3^, SE = 10.0, *p* = 0.24, total change =  − 4.7%).

The results of the per-protocol analysis (PP), (*n* = 7) comparing the outcome parameters between baseline and end of treatment, are shown in Table 2. Basically, these results mirror the results of the intention-to treat analyses. At the individual level in 6 out of 7 patients a lumbar spine BMD improvement above LSC was measured, while this was the case in 3/7 patients at femoral neck and at total hip. In per-protocol participants there was no significant increase in percent fat (*t* = − 1.83, df = 6, *p* = 0.12, change = 20.0%).

**Table 2:** Comparison of outcome parameters in the course of time for per-protocol participants (*n* = 7)

	Time baseline, mean (SD)	24 months, mean (SD)	Change	*t*-test *t* (df)	*p* (2-tailed)	Partial *η*^2^
Body mass index (kg/m^2^)	15.6 (1.7)	16.6 (1.9)	6.4%	− 1.96 (6)	0.10	0.39
Body composition: fat (%)	18.2 (5.0)	21.9 (4.9)	20.0%	− 1.83 (6)	0.12	0.36
Body composition: lean mass^a^ (kg)	33.4 (4.9)	34.7 (3.9)	4.0%	− 1.66 (6)	0.15	0.31
Bone mineral density						
Lumbar spine						
g/cm^2^	0.660 (0.102)	0.749 (0.084)	13.5%	− 3.79 (6)	< 0.01	0.71
* Z*-score	− 3.37 (0.98)	− 2.63 (0.79)				
Femoral neck						
g/cm^2^	0.565 (0.055)	0.593 (0.049)	4.9%	− 2.46 (6)	< 0.05	0.59
* Z*-score	− 2.53 (0.46)	− 2.26 (0.39)				
Hip total						
g/cm^2^	0.651 (0.049)	0.676 (0.053)	3.8%	− 1.70 (6)	0.14	0.32
* Z*-score	− 2.40 (0.39)	− 2.17 (0.40)				
Radius bone parameters						
Total bone density (mgHA/cm^3^)	253.6 (32.6)	245.5 (20.4)	− 3.2%	1.36 (6)	0.22	0.23
Cortical bone density (mgHA/cm^3^)	835.2 (58.9)	816.2 (65.6)	− 2.3%	3.53 (6)	< 0.05	0.67
Cortical thickness (μm)	565.7 (122.9)	534.3 (115.4)	− 5.6%	6.18 (6)	< 0.001	0.86
Trabecular bone density (mgHA/cm^3^)	134.6 (29.5)	132.4 (24.2)	− 1.6%	0.34 (6)	0.74	0.02
Meta trab. bone density (mgHA/cm^3^)	190.0 (29.0)	188.1 (22.7)	− 1.0%	0.38 (6)	0.72	0.02
Trabecular number (1/mm)	1.699 (0.301)	1.697 (0.360)	− 0.1%	0.04 (6)	0.97	0.00
Trabecular thickness (mm)	0.067 (0.018)	0.067 (0.017)	0.0%	0.00 (6)	1.00	0.00
Trabecular separation (mm)	0.539 (0.116)	0.554 (0.162)	2.6%	− 0.63 (6)	0.55	0.06
Tibia bone parameters						
Total bone density (mgHA/cm^3^)	261.4 (28.9)	250.0 (43.7)	− 4.3%	0.96 (5)	0.38	0.16
Cortical bone density (mgHA/cm^3^)	893.2 (37.3)	887.5 (48.8)	− 0.6%	1.06 (5)	0.34	0.18
Cortical thickness (μm)	888.3 (125.4)	883.3 (155.8)	− 0.6%	0.21 (5)	0.84	0.01
Trabecular bone density (mgHA/cm^3^)	154.1 (41.6)	141.7 (46.8)	− 8.0%	1.10 (5)	0.32	0.19
Meta trab. bone density (mgHA/cm^3^)	212.0 (39.0)	201.7 (43.5)	− 4.9%	1.02 (5)	0.35	0.17
Trabecular number (1/mm)	1.553 (0.374)	1.520 (0.456)	− 2.1%	0.62 (5)	0.56	0.07
Trabecular thickness (mm)	0.083 (0.018)	0.079 (0.017)	− 5.4%	1.23 (5)	0.27	0.23
Trabecular separation (mm)	0.595 (0.174)	0.643 (0.257)	7.9%	− 0.99 (5)	0.37	0.17

^a^Lean mass incl. bone mineral content

Analysis of the safety laboratory parameters of the per-protocol analysis revealed non-significant changes in many parameters over the course of time (see Table 3). However, vitamin D level decreased from baseline to the end of treatment (*t* = 3.26, df = 5, *p* < 0.05), as well as levels of parathyroid hormone (*t* = 2.53, df = 6, *p* < 0.05).

**Table 3:** Comparison of safety laboratory parameters in the course of time for per-protocol participants (n = 7)

	Time baseline, mean (SD)	24 months, mean (SD)	Change	*t*-test *t* (df)	*p* (2-tailed)	Partial *η*^2^
Calcium, total (mmol/l)	2.40 (0.09)	2.45 (0.08)	2.2%	− 0.97 (6)	0.37	0.14
Calcium albumin corr. (mmol/l)	2.25 (0.06)	2.29 (0.06)	1.8%	− 0.93 (6)	0.39	0.13
Phosphate (mmol/l)	1.15 (0.16)	1.12 (0.08)	− 2.7%	0.61 (5)	0.57	0.07
Creatinine (μmol/l)	58.1 (12.3)	60.6 (10.5)	4.2%	− 0.73 (6)	0.49	0.08
eGFR (crea) (ml/min)	119.7 (15.0)	116.7 (14.0)	− 2.5%	1.04 (6)	0.34	0.15
Vitamine D (μg/l)	42.7 (12.4)	34.5 (7.5)	− 19.2%	3.26 (5)	< 0.05	0.68
FSH (IE/l)	1.84 (4.19)	1.81 (2.26)	− 1.7%	0.03 (5)	0.98	0.00
Estradiol (pmol/l)	98.6 (150.9)	98.1 (106.8)	− 0.4%	0.01 (6)	1.00	0.00
Bone specific alkaline phosphatase (μg/l)	8.30 (2.35)	13.20 (7.80)	59.0%	− 1.53 (4)	0.20	0.37
Parathyroid hormone, intact (ng/l)	41.9 (11.9)	24.2 (10.7)	− 42.2%	2.53 (6)	< 0.05	0.52

We did not observe any fracture in the patients during the overall study period.

### Adverse Effects

TPT was generally well-tolerated by all study participants. Five patients reported nausea, two suffered from headache, two from dizziness. Rarely some patients reported pain in the thigh, or paresthesia of the arms. All this effects mostly presented in the first 2 weeks of the treatment, no patient discontinued the treatment due to side effects.

## Discussion

The aim of this pilot study was to evaluate the effect of a 24-month treatment with TPTH in a small group of AN patients with low BMD and increased bone fragility. The working hypothesis was that TPT could improve bone mineral density and the structure of young severely ill anorexic patients.

After 24 months of treatment with the anabolic agent TPT, the BMD increased significantly in the spine 13.5% and in the femoral neck 5.0% in young women with severe AN. This is a strong increase, in comparison with other substances [6]. Our results showed that TPT mediated a strong improvement of BMD in a group of young and severe underweight ill patients. It is noteworthy that adverse effects of the drug were minimal, which demonstrated that TPT represents a safe and effective treatment for AN patients with very low BMD. Important is also the fact that during the observed 2 years no clinical significant fractures occurred.

Since normalization of the body weight has a positive effect on bone BMD [6], in this context, the development of the patients’ BMI during the study is important. During the course of the study, patients’ BMI did not increase significantly, while the percent of body fat did. However, after controlling these potential confounders, our results were not altered unfavorably. Thus, the effect of TPT on BMD seems to be positive, regardless of a parallel body weight gain. In this context, it is cogitable that the intake of sexual hormones may have contributed to the positive results of the BMD.

To our knowledge, there is only one study that investigates the effect of TPT in very young AN patients. Fazeli et al. [13] administer the same substance during only 6 months. Also this study showed a positive effect of BMD, but it was assessed in a remarkably older population (aged 47 years ± 2.7; our sample age 25 ± 4.4 years) than in our study. The two studies show other differences: the sample of Fazeli et al. was not severe underweight (mean BMI 17.6 ± 0.4 kg/m^2^), compared with our sample (mean BMI 15.6 ± 1.6 kg/m^2^). In addition, the results of the two studies are not identical. Fazeli et al. reported an increase of BMD only in the spine, and not in other localizations. We could find a very strong increase of the bone density of the spine; in addition,—and contrary to Fazeli et al. we could find also a significant increase of the bone density of the femoral neck. This result underlines the importance of a longer duration of the TPT treatment than 6 months (24 months), and have to be verified in larger studies. Since our sample was younger, it is also possible that the bone regeneration potential at a younger age is better than in premenopausal women, like in the study of Fazeli et al. As far as we know, this is the first study that analyzes the effect of TPT with HRpqCT. Regarding the HRpqCT measurement of the bone microarchitecture of the radius, we could find an interesting reduction in the cortical thickness after 2 years, while the trabecular bone density (trabecular number and trabecular thickness) seems to remain constant. Remarkably different are the results of the tibia, indeed the bone architecture under TPT-therapy seems to be spared from significant negative changes when we do not consider the cortical thickness which becomes slightly thinner, and the trabecular number which decreases slightly. The lack of amelioration of the HRpqCT parameters of the bone microarchitecture under TPT at the end of the treatment underlines the well-known difficulty of regeneration of the peripheral bone in AN [17], so that in these patients a stabilization of bone parameters—a non-deterioration—could be interpreted as a positive result.

Our results (and the study results of Fazeli et al.) could suggest that the positive effect of TPT would be related more to the proximal bone and less to the peripheral bone.

The differences of the bone microarchitecture parameters of the two peripheral bones (radius and tibia) may in this sample—AN patients move willingly until becoming hyperkinetic—be explained by a different exposure to the weight-bearing activity; in effect, tibia is much more exposed to repetitive weight-bearing activity than radius [18].

Unfortunately, in the present prospective study, we did not have a comparison (placebo) group. Several studies underline the negative course of the BMD in AN patients with lack of weight gain [6]. As this was an uncontrolled, one-arm pilot study, our results are limited to observations occurring in the whole group of participants over time. However, it can be assumed that this patient-group—with negative weight course—would not have had any positive bones course without pharmacological intervention. Regrettably is the reduction of the sample (three patients) during the study, however dropouts and reduced compliance are well-known in severe AN patients.

It is an important question if there will be a BMD loss after stopping TPT treatment in this young sample. Because a subsequent measurement after 2 years of treatment was not undertaken in this study, this is an issue for future research.

Low BMD is a very common medical comorbidity associated with AN. Notably, bone loss is associated with an increased risk of fracture, and with this a low quality of life.

## Conclusion

AN is an illness which is difficult to treat, with patients often showing a chronical course. The starvation state leads to changes in many hormonal systems and can lead to low BMD which is an important negative consequence of AN. The therapeutic possibilities against low BMD and increased bone fragility are limited. TPT treatment over 24 months might be a viable and safe option to treat severe young patients with little chances to gain weight. Larger controlled studies should be conducted to better test the efficacy and tolerance of this substance.
